# The metal chaperone protein MtmA plays important roles in antifungal drug susceptibility in *Aspergillus fumigatus*

**DOI:** 10.3389/fmicb.2022.1062282

**Published:** 2022-12-02

**Authors:** Pengfei Zhai, Yinyan Ma, Wenlong Du, Ling Lu

**Affiliations:** ^1^Jiangsu Key Laboratory for Microbes and Functional Genomics, College of Life Sciences, Nanjing Normal University, Nanjing, China; ^2^Department of Biophysics, College of Life Sciences, Xuzhou Medical University, Xuzhou, Jiangsu, China

**Keywords:** *Aspergillus fumigatus*, MtmA, mitochondria, azole, multidrug resistance

## Abstract

Drug-resistant fungal infections are emerging as an important clinical problem. In general, antifungal resistance results from increased target expression or mutations within the target protein sequence. However, the molecular mechanisms of non-drug target mutations of antifungal resistance in fungal pathogens remain to be explored. Previous studies indicated that the metal chaperone protein Mtm1 is required for mitochondrial Sod2 activation and responses to oxidative stress in yeast and in the fungal pathogen *Aspergillus fumigatus*, but there is no report of MtmA-related antifungal resistance. In this study, we found that repressed expression of MtmA (only 10% expression) using a conditional promoter resulted in significantly enhanced itraconazole resistance, which was not the result of highly expressed drug targets Erg11A and Erg11B. Furthermore, we demonstrated that repressed expression of MtmA results in upregulation of a series of multidrug resistance-associated transport genes, which may cause multidrug resistance. Further mechanistic studies revealed that inhibition of MtmA expression led to abnormal activation of the calcium signaling system and prompted persistent nucleation of the calcium signaling transcription factor CrzA. Our findings suggest that the metal chaperone protein MtmA is able to negatively regulate fungal resistance *via* affecting calcium signaling pathway.

## Introduction

*Aspergillus fumigatus* is an opportunistic pathogenic fungus that is widespread in the natural environment and can cause severe invasive aspergillosis in immunodeficient hosts (van de Veerdonk et al., 2017; Arias et al., 2018; Lima et al., 2019). Due to the wide use of immunosuppressants, the number of patients with immune deficiency is on the rise, which leads to the increasing number of invasive aspergillosis cases caused by *A. fumigatus* infection (Roemer and Krysan, 2014; Verweij et al., 2016). To date, the commonly used antifungal drugs include azoles, allylamines, polyenes and echinocandins (Valiante et al., 2015; Robbins et al., 2017). Because of their high efficacy and less side effects, azoles are recommended as first-line drugs in the treatment of fungal infections (Roemer and Krysan, 2014; Denning and Bromley, 2015). Azoles inhibit ergosterol biosynthesis and accumulate toxic intermediates mainly through specific inhibition of the lanosterol 14-α demethylase Erg11 (also referred to as Cyp51), which ultimately leads to fungal cell death (Ferreira et al., 2005; Perez-Cantero et al., 2020). The massive use of azole drugs in clinical therapy and agricultural production has led to a significant increase in antifungal-resistant fungal pathogens, posing a new challenge for the treatment of invasive aspergillosis (Camps et al., 2012; Chowdhary et al., 2013; Verweij et al., 2020). Antifungal resistance refers to the ability to grow at antifungal drug concentrations above established antifungal susceptibility breakpoints, usually (but not exclusively) following adaptation to antifungal drug exposure resulting in established causal molecular changes (Berman and Krysan, 2020; Fisher et al., 2022). This is expressed as the minimum inhibitory concentration (MIC). According to previous studies, there are three main mechanisms of azole resistance in pathogenic fungi: the mutation of the drug target Erg11, the upregulation of drug efflux pumps and the involvement of cellular stress response pathways (Ferreira et al., 2004; Cowen et al., 2009; van der Linden et al., 2011; Liu et al., 2015; Li et al., 2020). The most common mechanism of azole resistance in *A. fumigatus* is mutation of the drug target Erg11A or its promoter. Mutations in some sites of Erg11A reduce the affinity of azoles, and some mutations in its promoter region result in increased Erg11A transcription, leading to azole resistance (Snelders et al., 2008; Verweij et al., 2009; Mohammadi et al., 2018). Currently, two main types of drug pumps have been reported to be involved in drug resistance: the major facilitator superfamily (MFS) and the ATP-binding cassette (ABC) transporter, both of which are capable of transporting antifungal drugs out of the cell to reduce drug accumulation in fungal cells, thereby leading to azole resistance (Ferreira et al., 2004; Cannon et al., 2009; Sturm et al., 2020).

Many studies have focused on azole resistance caused by Erg11A mutations and high expression of drug pumps in clinical and environmental settings (Bueid et al., 2010; Perez-Cantero et al., 2020). In recent years, an increasing number of *A. fumigatus* resistant isolates have been identified for having non-*cyp51A* mutations but with unexplored verification related to drug resistance (Ener et al., 2022). The integrity of mitochondrial function is an important guarantee of multiple cellular processes (Vakifahmetoglu-Norberg et al., 2017; Akbari et al., 2019; Rossi et al., 2019). Recent studies have shown that pathogenic fungi adopt an adaptive strategy to reduce mitochondrial metabolic function in response to antifungal drug adversity (Li et al., 2020). Moreover, studies have shown that the expression of mitochondria-related genes is closely associated with the resistance of pathogenic fungi to antifungal azoles (Neubauer et al., 2015; Li et al., 2020; Zhou et al., 2022). Mitochondria are involved not only in many biological processes as energy factories but also as intracellular Ca^2+^ stores capable of participating in the response to external stimuli (Ganitkevich, 2003). The Ca^2+^-mediated signaling pathway is capable of responding to stimulation by antifungal drugs such as itraconazole, which is able to induce a sharp increase in cytoplasmic free Ca^2+^ (Juvvadi et al., 2015; Li et al., 2019; Zeng et al., 2019). Previous studies have found that the most critical transcriptional regulator of the Ca^2+^-mediated signaling pathway, CrzA, shifts from the cytoplasm to the nucleus when the fungus encounters drugs or other adverse conditions, inducing the upregulation of drug pumps such as *mdr1* (Juvvadi and Steinbach, 2015; Liu et al., 2015; Shwab et al., 2019; Zeng et al., 2019; Li et al., 2020). However, it is not fully known which functional genes in the mitochondria of pathogenic fungal cells may be involved in drug resistance. Our previous studies have shown that the mitochondria-localized metal chaperone protein MtmA is required for mitochondrial function, the oxidative stress response and hyphal growth (Zhai et al., 2022). In yeast, Mtm1 (homologs of MtmA) has recently been found to translocate pyridoxal 5′-phosphate (PLP) into mitochondria, thereby controlling PLP-dependent activities such as heme biosynthesis, FeS cluster biosynthesis, and iron homeostasis but this has not been reported yet in *A. fumigatus* (Park et al., 2013; Whittaker et al., 2015). In the present study, we found that a novel function of the metal chaperone protein MtmA is its involvement in the susceptibility of *A. fumigatus* to a diversity of antifungal drugs. By using a conditional promoter to turn off MtmA expression (approximately 10% expression), it was found to significantly increase the resistance of the strain to a variety of antifungal drugs. Further mechanistic studies showed that reduced MtmA expression leads to abnormal activation of the Ca^2+^ signaling system involved in the response to stress, resulting in persistent nucleation of the transcription factor CrzA and thus abnormally high expression of the drug efflux pump involved in the drug response. Our study shows that mitochondria-localized MtmA is critical for antifungal drug resistance involving drug efflux pumps that depend on the Ca^2+^ signaling system.

## Materials and methods

### Strains, media and culture

All *A. fumigatus* strains and primers used in this study are listed in Supplementary Tables S1, S2, respectively. The strain A1160 was purchased from the Fungal Genetics Stock Center. Minimal medium: MM + glucose (containing 1% glucose, trace elements, and 50 ml liter^−1^ of 20x salt, pH 6.5), MM + glycerol (containing 1% glycerol, trace elements, and 50 ml liter^−1^ of 20x salt, pH 6.5) and YAG medium (containing 2% glucose, 0.5% yeast extract, and 1 ml/l 1,000x trace elements). For colony morphology analysis, all strains were grown on YAG media at 37°C for 1.5 d to 2 d. The *A. fumigatus* transformation method was performed according to previous references (May, 1989; Sanchez et al., 1998).

### Construction of GFP labeling strains

For the construction of GFP-tagged strains, we used a homologous recombination approach in which the stop codon of the target gene was replaced with a fusion fragment of GFP + *pyrG*. We transferred plasmids containing the Erg11A::GFP, Erg11B::GFP (Song et al., 2016a) and CrzA::GFP (Li et al., 2020) cassettes into the *P_alcA_::mtmA* strain by transformation to produce the *P_alcA_::mtmA*^Erg11A::GFP^, *P_alcA_::mtmA*^Erg11B::GFP^ and *P_alcA_::mtmA*^CrzA::GFP^ strains, respectively.

### Western blotting

To determine the protein expression levels of Erg11A and Erg11B, 5 × 10^7^ spores of the related strains were inoculated into liquid MM and shaken at 200 rpm for 24 h at 37°C. The obtained mycelium was ground into powder with liquid nitrogen and then extracted with ice-cold extraction buffer (1 μg ml^−1^ leupeptin, 1 mM EDTA, 137 mM KCl, 1 μg ml^−1^ pepstatin A, 1 mM PMSF, 50 mM HEPES and 10% glycerol, pH 7.4). The specific method of western blotting was performed according to a previous study (Zhai et al., 2019).

### Ergosterol extraction and analysis

For the extraction and analysis of ergosterol, 5 × 10^7^ conidia were inoculated into 100 ml of MM liquid medium and incubated at 37°C and 220 rpm for 10 h, followed by the addition of 0.05 μg ml^−1^ ITC and continued incubation for 14 h. The mycelia were collected and washed three times with distilled water and then freeze-dried and weighed. Approximately 100 mg of dry mycelia were mixed with 3 ml 25% ethanol potassium hydroxide solution (2:3, alcohol to methanol) and then vortexed for 1 min. After incubated 1 h at 85°C, mycelia were harvested with 3 ml hexane and 1 ml distilled water and then were vortexed for 3 min. The upper hexane layer was transferred to another tube after the tube was placed at room temperature for 10 min and evaporated to near dry in a fume hood. Next, all the dried samples were dissolved with 1 ml methanol and filtered using 0.2 μm pore size filters. Total ergosterol was analyzed using HPLC on an AQ-C18 column (250 mm by 4.6 mm, 5 μM) with a methanol flow rate of 1 ml min^−1^, 280 nm as the detection wavelength (Zhai et al., 2019).

### Measurement of R6G uptake and glucose-induced efflux abilities

The intracellular concentration of R6G (Sigma) in *A. fumigatus* was evaluated as described previously (Wei et al., 2017). Approximately 5 × 10^6^ fresh conidia were grown in YAG medium at 37°C until conidia began to germinate. The samples in culture dishes were washed and then suspended in phosphate-buffered saline (PBS), to which R6G was added at a final concentration of 10 μM, and the mixtures were incubated at 37°C for 1 h. The samples were then washed with PBS, and the fluorescence of R6G in samples was measured by fluorescence-activated cell sorter (FACS) analysis (Accuri C6; BD). To assess energy-dependent R6G efflux, the samples were suspended in PBS containing 1 M glucose and incubated for 0.5 h at 37°C. After the treated conidia had been washed twice with PBS, the fluorescence intensity of samples was measured by FACS analysis.

### Cellular azole drug retention

For the detection of intracellular ITC retention, 5 × 10^7^ conidia were inoculated into 100 ml of MM + glucose liquid medium and incubated at 37°C and 220 rpm for 23 h. Then, 1 μg ml^−1^ ITC was added, and incubation was continued for 1 h. The mycelia were collected, washed three times with distilled water, freeze-dried and weighed. Approximately 50 mg of mycelium powder was weighed, and ITC extraction and analysis were performed as previously described (Wei et al., 2017; Zhai et al., 2019).

### Fluorescence microscopy

Fresh conidia of related strains expressing CrzA-GFP were incubated in 2 ml of liquid MM + glucose by different treatments (see legend) at 37°C for the indicated times. Next, the medium was pipetted away and washed three times with phosphate-buffered saline (PBS). Then, the samples were fixed with 4% (vol/vol) formaldehyde for 30 min at room temperature and washed three times with PBS. To observe the nuclei, the mycelia were stained with a final concentration of 100 mg ml^−1^ Hoechst 33528 for 30 min. Images were captured using a Zeiss Axio Imager A1 microscope (Carl Zeiss, Jena, Germany).

### RNA-seq analysis and RT-PCR

For RNA-seq analysis, 5 × 10^7^ conidia of the *P_alcA_::mtmA* strain and WT strain were incubated in liquid MM + glucose in a rotary shaker at 220 rpm at 37°C for 24 h. After incubation, mycelium was filtered and immediately harvested in liquid nitrogen. After mRNA extraction, purification, and library construction, sequencing was performed by next-generation sequencing (NGS) based on the Illumina sequencing platform. A fold change of ≥2 and a *p* value of <0.05 were set as the threshold values for differentially expressed genes. All samples were prepared to perform digital transcriptome analyses through the RNA-seq approach (Shanghai Sangon Biotech Co., Ltd., China). For RT-PCR, 5 × 10^7^ conidia of the *P_alcA_::mtmA* strain and its parental wild-type (WT) strains were incubated in liquid MM + glucose for 24 h at 37°C. The obtained mycelia were ground into powder with liquid nitrogen, and total RNA was isolated using the UNlQ-10 Column TRIzol total RNA isolation kit (Sangon Biotech, B511361) according to the manufacturer’s directions. RNA was reverse transcribed into cDNA with a HiScript III RT SuperMix kit, and the resulting cDNA was used for transcription detection. RT–PCR analysis was performed as described in previous studies (Zhai et al., 2019).

### Measurement of the free Ca^2+^ content

For measurement of cytoplasmic calcium transients, the plasmids containing pAEQcyt were transformed into related *A. fumigatus* strains. Fresh 10^6^ spores expressing aequorin were inoculated in 100 μl MM + glucose of each well of a 96-well microtiter plate (Thermo Fisher) and incubated for 18 h at 37°C. Six wells were used in parallel for each treatment. The medium was then removed from each well and washed twice with PGM (50 mM glucose, 1 mM MgCl_2_, 20 mM PIPES pH 6.7). Aequorin was reconstituted by incubating mycelia in 100 μl PGM with 2.5 μM coelenterazine f (Sigma-Aldrich) for 4 h at 4°C in the dark. After that, the mycelium was washed twice with 200 μl of PGM and incubated at room temperature for 1 h. At the end of each experiment, active aequorin was completely discharged by permeabilizing the cells with 20% (vol/vol) ethanol in the presence of excess Ca^2+^ (3 M CaCl_2_) to determine the total aequorin luminescence of each culture. Luminescence was measured on an LB 96P Microlumat Luminometer (Berthold Technologies, Germany), which was controlled by a dedicated computer running the Microsoft Windows-based Berthold WinGlow software. The mathematical conversion of luminescence values (relative luminescence units [RLUs]) into [Ca^2+^]_c_ concentrations was carried out as described previously (Nelson et al., 2004; Zhang et al., 2016). Samples were analyzed from three independent biological replicates.

### β-Galactosidase assays

We transformed the plasmid containing the *mdr1(p)::lacZ* cassette into the WT and *P_alcA_::mtmA* to generate WT*^mdr1(p)::lacZ^* and *P_alcA_::mtmA^mdr1(p)::lacZ^*, respectively. A total of 5 × 10^7^ spores of the related strains were inoculated into liquid MM + glucose and shaken at 200 rpm for 24 h at 37°C. The obtained mycelia were ground into powder with liquid nitrogen. The specific method of β-galactosidase assays was performed according to a previous study (Du et al., 2021).

## Results

### Repressed expression of MtmA results in enhanced itraconazole resistance

**Figure 1:**
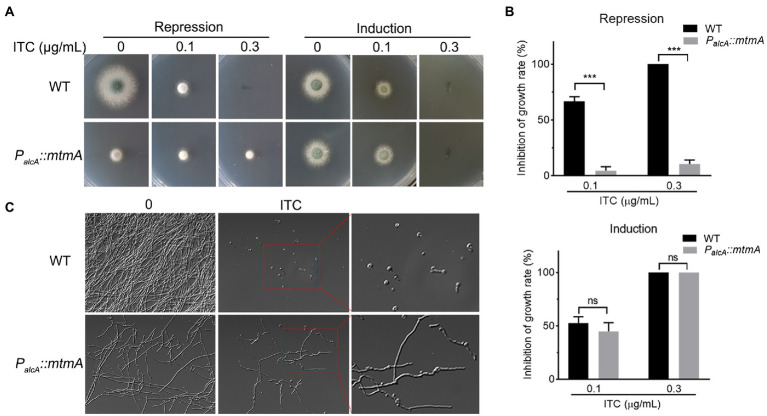
Repressed expression of MtmA causes itraconazole resistance. **(A)** Conidia of each indicated strain were inoculated on repression (MM + glucose) or induction (MM + glycerol) medium containing serial concentrations of itraconazole (ITC) for 2 days at 37°C. **(B)** Mycelium growth inhibition ratios of the indicated strains on YAG and MM plus glycerol with different concentrations of itraconazole (ITC). Data are presented as the means ± standard deviation (SD) of the results from three independent experiments. Statistical significance was determined by Student’s *t* test. ***, *p* < 0.001; ns, not significant. **(C)** Hyphal morphologies of the indicated strains grown on liquid MM + glucose in the presence or absence of 0.5 μg/ml ITC for 24 h at 37°C.

To characterize the relationship between mitochondria-localized MtmA and azole antifungals, we used the previously constructed conditional strain *P_alcA_::mtmA* (approximately 10% mRNA expression of *mtmA* in MM + glucose repression medium) to further explore the function of MtmA (Zhai et al., 2022). The alcohol dehydrogenase promoter (*alcA*)-controlled *P_alcA_::mtmA* strain was induced with ethanol or threonine and derepressed by glycerol but repressed with glucose as a carbon source. As shown in Figures 1A,B and Supplementary Figure S1, the *P_alcA_::mtmA* mutant showed enhanced resistance to media supplemented with itraconazole (ITC) under repression conditions compared to the wild-type (WT) strain, while no difference was found in the induction conditions, suggesting that reduced expression of *mtmA* increased itraconazole resistance. Moreover, this resistance phenomenon was consistently found in the liquid repression medium after the addition of itraconazole (Figure 1C). Taken together, these data suggest that MtmA is required for itraconazole susceptibility in *A. fumigatus*.

### MtmA affects the expression of the azole antifungal drug target Erg11

**Figure 2:**
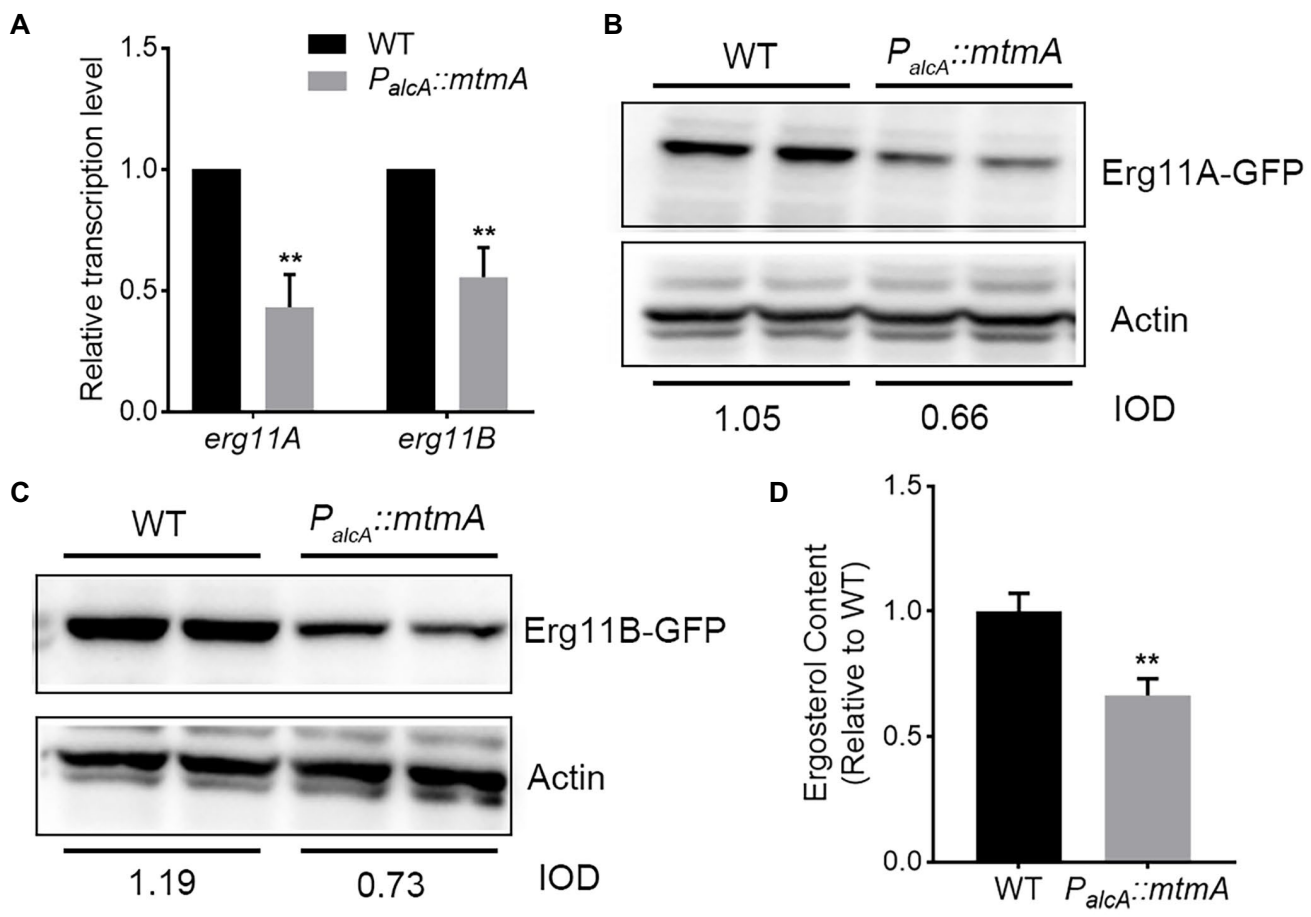
MtmA is required for the expression of Erg11A/B. **(A)** The transcript levels of the indicated genes in WT and the *mtmA*-repressed strains grown at 37°C for 24 h under MM + glucose inhibition culture conditions. Error bars indicate the mean ± SD of the results from three independent experiments. Statistical significance was determined by Student’s *t* test. **, *p* < 0.01. **(B,C)** Western blot analysis of the expression of Erg11A and Erg11B in the parental wild-type and *mtmA*-repressed strains under MM + glucose inhibition culture conditions. Actin was used as the loading control. The value of the integrated optical density (IOD; IODtarget protein/IODactin) measured by ImageJ indicates the relative protein quantity. **(D)** The same number of spores from the indicated strains grown for 24 h at 37°C in MM + glucose with 0.02 μg/ml ITC for subsequent ergosterol measurement by HPLC. Values indicate the mean ± SD of the results from three independent experiments. Statistical significance was determined by Student’s *t* test. **, *p* < 0.01.

Enhanced expression of Erg11, a direct target of azole antifungal drugs, is thought to be an important reason for fungal resistance to azole drugs (Blatzer et al., 2011; Gsaller et al., 2016). To investigate whether the resistance of the *P_alcA_::mtmA* strain to the antifungal drug itraconazole is due to increased expression levels of the azole drug target Erg11, we determined the transcript levels of *erg11A* and *erg11B* by RT–PCR. Unexpectedly, the RT–PCR results showed that repression of MtmA expression significantly reduced the transcript levels of *erg11A* and *erg11B* (Figure 2A). To further verify the protein expression levels of Erg11A and Erg11B, we constructed two GFP-tagged C-terminal Erg11A and Erg11B strains in the *P_alcA_::mtmA* background. As shown in Figures 2B,C, western blotting results showed that repression of *mtmA* expression significantly reduced the expression of Erg11A and Erg11B relative to the WT strain. These data suggest that MtmA is required for Erg11A and Erg11B expression. Moreover, we used high-performance liquid chromatography (HPLC) to determine the ergosterol content. Consequently, under itraconazole treatment conditions, the ergosterol content of the *P_alcA_::mtmA* mutant was reduced by approximately 35% compared to that of the WT strain (Figure 2D). Together, these data suggest that enhanced itraconazole resistance in the conditional *mtmA*-repressed strain is not the result of highly expressed drug targets Erg11A and Erg11B.

### Repressed expression of MtmA results in upregulation of a series of multidrug resistance-associated transport genes

**Figure 3:**
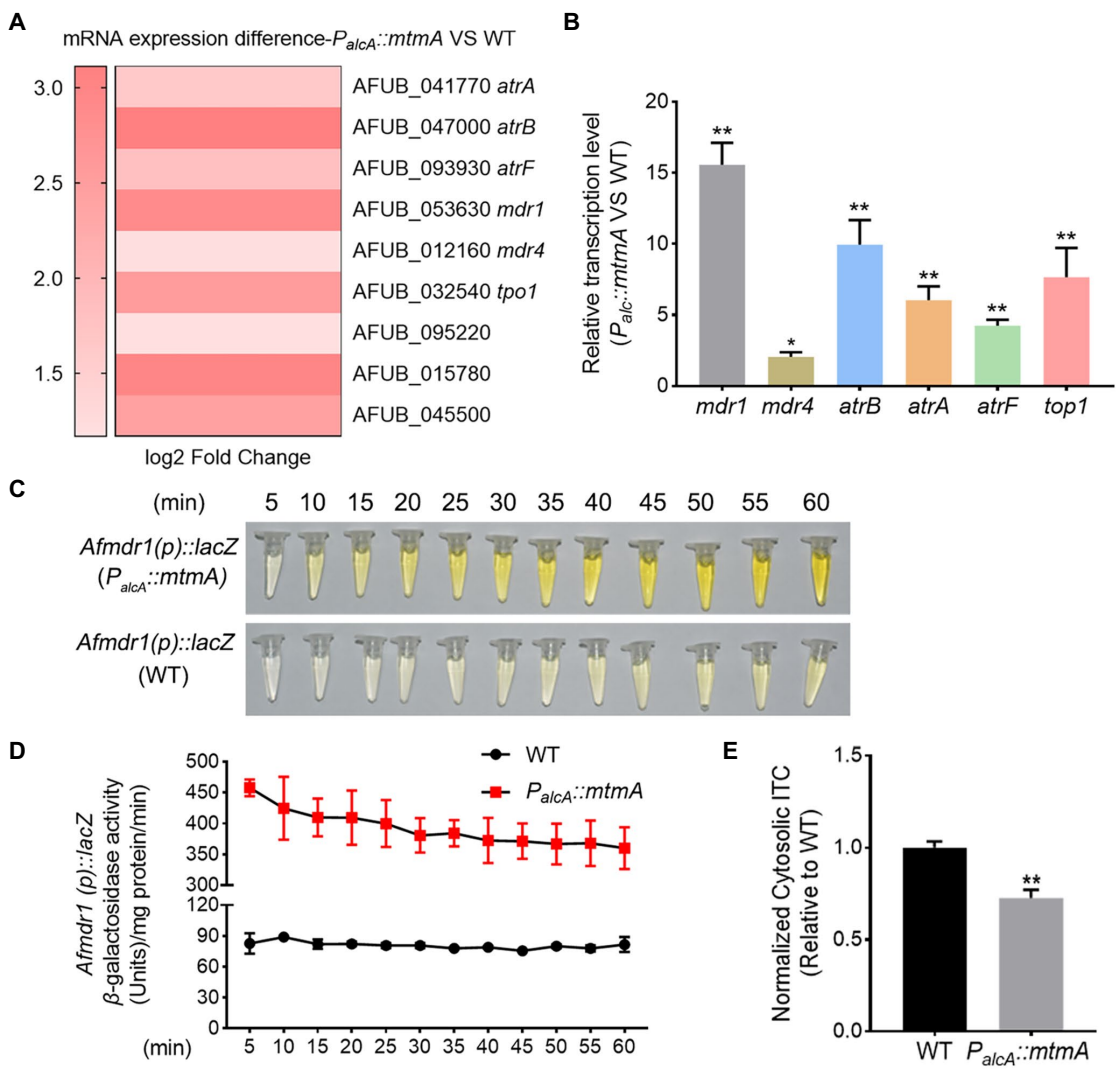
Repressed expression of MtmA induces upregulation of a series of drug resistance-related transport genes. **(A)** Heatmap of RNA-seq data for the 9 selected genes. **(B)** The transcript levels of the indicated genes in WT and the *mtmA*-repressed strains grown at 37°C for 24 h. Error bars indicate the mean ± SD of the results from three independent experiments. Statistical significance was determined by Student’s *t* test. *, *p* < 0.05; **, *p* < 0.01. **(C,D)** The transcript level of the multidrug-resistant transport gene *mdr1* was confirmed by a β-galactosidase activity assay. Error bars indicate the mean ± SD of the results from three independent experiments. **(E)** The intracellular ITC concentrations in the indicated strains analyzed by HPLC. Normalized quantification and comparison of the ITC contents in WT and the *mtmA*-repressed strains. Values indicate the mean ± SD of the results from three independent experiments. **, *p* < 0.01.

To further explore the molecular mechanisms involved in drug resistance by MtmA, we identified differentially expressed genes between the *mtmA*-repressed strain and WT strain by transcriptome profiling experiments (RNA-seq). The results showed that a series of differentially expressed genes, including 106 downregulation genes and 626 upregulation genes in the *mtmA*-repressed strain compared to the WT strain(adjusted *p*-value ≤0.05, |log_2_FC| ≥ 1) (Dataset 1). Gene Ontology (GO) classification analysis showed that the amounts of proteins involved in transmembrane transport were enriched (Supplementary Figure S4). Further enrichment analysis of signaling pathways using the KEGG database showed that the aforementioned differentially expressed genes were mainly mapped to drug metabolism processes (Supplementary Figure S5). As shown in Figure 3A, we found that two types of fungal drug efflux pumps, ATP binding cassette (ABC) transporters and the major facilitator superfamily (MFS), were significantly upregulated. To further verify whether the expression of these genes could be different between the *mtmA*-repressed strain and WT strain, RT–PCR was carried out to examine the expression of the relevant genes. As shown in Figure 3B, RT–PCR analysis of the expression of these six genes was highly consistent with the RNA-seq data, suggesting that MtmA may be involved in drug susceptibility by affecting the expression of drug transporters. To further visualize the differential expression between the *mtmA*-repressed strain and the WT strain, we used *mdr1* as a representative by fusing the *mdr1* promoter with a bacterial *lacZ* reporter gene. The *mdr1* gene is a key drug efflux pump gene that actively excretes multiple drugs from the intracellular environment. As shown in Figures 3C,D, the β-galactosidase activity increased approximately 5-fold, and samples extracted from the *mtmA*-repressed strain showed a yellow color compared to samples from the WT strain, suggesting that the promoter of *mdr1* is indeed more active in the *mtmA*-repressed strain. Next, to confirm whether these drug efflux pump genes reduced the retention of antifungal drugs in the *mtmA*-repressed strain, we used the fluorescent dye rhodamine 6G (R6G), as a mimicking substrate of ATP-binding cassette (ABC) transporters. The results showed that the retention rate of R6G in the *mtmA*-repressed strain was significantly lower than that of the parental wild-type strain (Supplementary Figure S2). To assess whether the retention of R6G in *mtmA*-repressed strain mimics the accumulation of antifungal drugs, we directly measured the intracellular ITC content using high-performance liquid chromatography (HPLC) analysis. As shown in Figure 3E, the retention of ITC in the *mtmA*-repressed strain was significantly lower than that in the WT strain. These data suggest that antifungal drug resistance caused by repression of *mtmA* expression might be mainly due to significant upregulation of the drug efflux pump.

**Figure 4:**
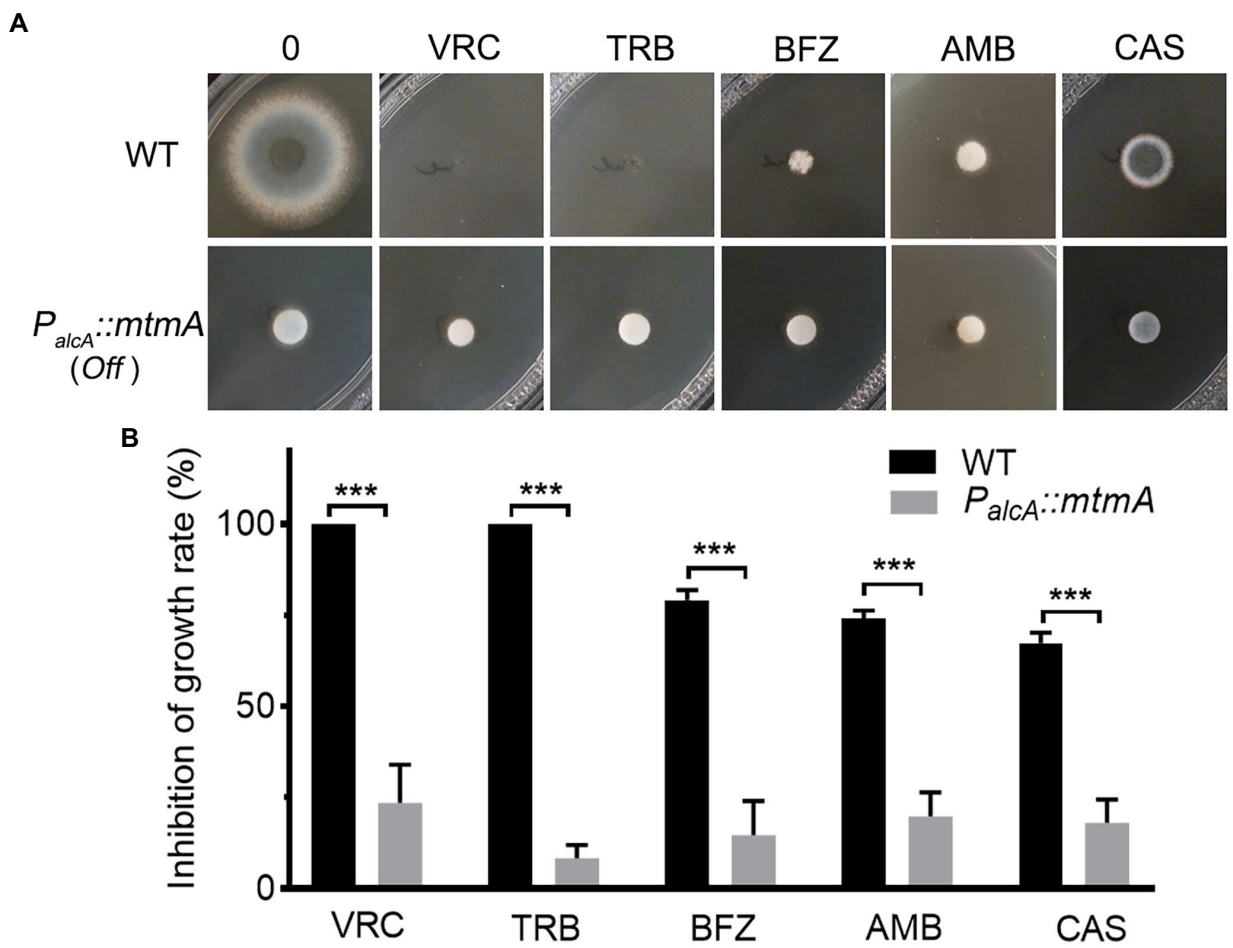
Repressed expression of MtmA causes multidrug resistance. **(A)** Colony morphology of the indicated strains inoculated on YAG medium without drug at 37°C for 1.5 days or with drug at 37°C for 2 days. The following antifungal drugs were used: 0.2 μg/ml voriconazole (VRC), 0.6 μg/ml terbinafine (TRB), 1.2 μg/ml bifonazole (BFZ), 3 μg/ml amphotericin B (AMB) and 0.5 μg/ml caspofungin (CAS). **(B)** Mycelium growth inhibition ratios of the indicated strains with different concentrations of the indicated drugs. Error bars indicate the mean ± SD of the results from three independent experiments. Statistical significance was determined by Student’s *t* test. ***, *p* < 0.001.

### Repressed expression of MtmA causes multidrug resistance to azoles, terbinafine, amphotericin B and caspofungin

The aforementioned data suggest that repressed expression of *mtmA* leads to upregulation of a series of drug efflux pump genes. We next tested the growth phenotypes of the related strains under different types of antifungal drugs, including azoles (voriconazole, bifonazole targeted for 14-α-lanosterol demethylase Erg11) (Ferreira et al., 2005), and polyenes (amphotericin B which not only kill cells by binding ergosterol to form pores and disrupt the integrity of cell membranes, but also damage cells by inducing oxidative stress; Zhai et al., 2019), allylamines (terbinafine targeted for squalene epoxidase Erg1; Ferreira et al., 2005), and echinocandins (caspofungin targeted for β-1,3-glucan synthase; Lima et al., 2019). Equal numbers of conidia from the parental wild-type and the *mtmA*-repressed strains were spotted onto YAG medium under treatment with different antifungal drugs. As shown in Figures 4A,B, under the repression condition, the *P_alcA_::mtmA* strain significantly increased resistance to voriconazole (VRC), bifonazole (BFZ), terbinafine (TRB), amphotericin B (AMB), and caspofungin (CAS) compared to the WT strain. These results suggest that reduced expression of MtmA induces resistance to multiple antifungal drugs in *Aspergillus fumigatus*.

### Repressed expression of MtmA causes persistent nuclear localization of CrzA, which contributes to drug resistance

**Figure 5:**
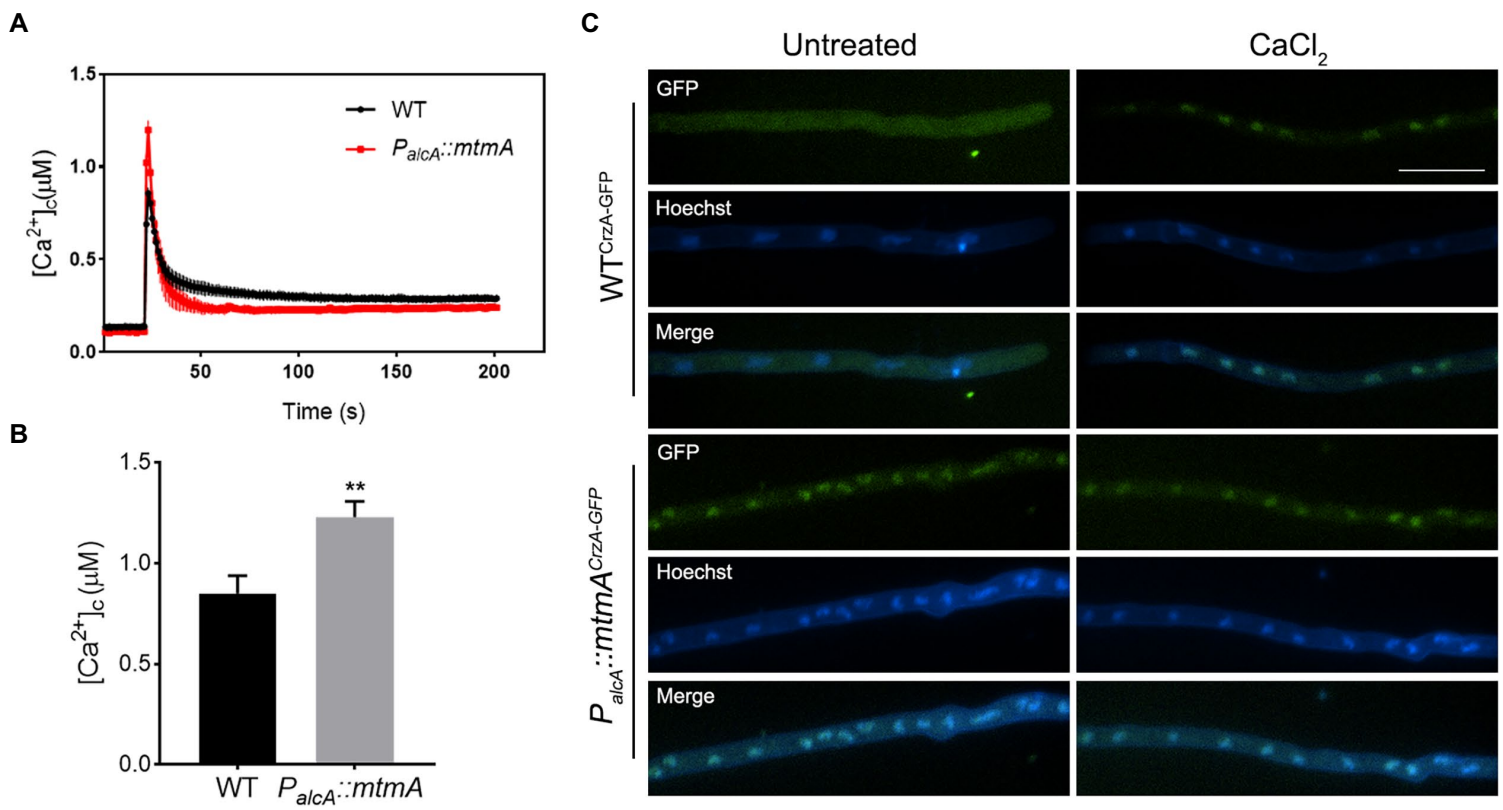
Repressed expression of MtmA led to enhanced transient cytosolic Ca^2+^ levels and localization of CrzA-GFP. **(A,B)** The aequorin-expressing strains were stimulated with 100 mM CaCl_2_ after growth in MM + glucose. The linear graphs indicated that the real-time [Ca^2+^]_c_ changes in response to calcium stimuli. [Ca^2+^]_c_, free Ca^2+^ concentration in the cytoplasm. Basal [Ca^2+^]_c_ was indicated by the resting level prior to extracellular calcium stimulus at the 50-s time point. [Ca^2+^]_c_ amplitude was indicated by the poststimulatory peak value of [Ca^2+^]_c_. Data are the average from at least six experiments. Values represent the mean ± SD. **, *p* < 0.01. **(C)** Epifluorescence microscopic images demonstrating the CrzA-GFP distribution under untreated or treatment with CaCl_2_ (100 mM) for 15 min in the WT and the *mtmA*-repressed strains. Hoechst is a nuclear localization signal dye used to visualize the nucleus. The scale bar represents 10 μm.

To investigate the possible mechanism by which the repression of *mtmA* could affect drug pump upregulation, we hypothesized that this drug resistance phenomenon induced by reduced expression of mitochondria-localized MtmA might be related to the dysregulation of fungal mitochondrial function. Since many lines of evidence have shown that calcium buffering by mitochondria is a key feature in cell survival (Ganitkevich, 2003; Song et al., 2016b), we next wondered whether the involvement of MtmA in multidrug susceptibility was associated with the calcium signaling pathway. To test this hypothesis, we constructed relevant strains containing aequorin for real-time monitoring of the dynamics of free cytoplasmic Ca^2+^ ([Ca^2+^]_c_) in live mycelial cells. As shown in Figures 5A,B, the [Ca^2+^]_c_ amplitude was significantly increased in the *mtmA*-repressed strain compared to the WT strain, indicating that the repressed expression of *mtmA* resulted in a significantly higher cytosolic Ca^2+^ capacity than the WT strain, suggesting that the repression of *mtmA* truly shapes cytosolic calcium signaling. Previous reports have shown that increased cytoplasmic free Ca^2+^ can lead to nuclear translocation activation of the transcription factor CrzA (Shwab et al., 2019; Li et al., 2020; Ren et al., 2021). Therefore, we constructed C-terminal GFP-tagged CrzA (CrzA-GFP) strains in the background of WT and *P_alcA_::mtmA* strains. As shown in Figure 5C, CrzA-GFP was mainly located in the cytoplasm of the WT strain when cultured in minimal medium. However, when CaCl_2_ was added to the medium, almost all of the CrzA-GFP was localized in the nucleus. In comparison, CrzA-GFP was consistently localized in the nucleus regardless of calcium stimulation in the *mtmA*-repressed strain, suggesting that repression of MtmA expression resulted in sustained nuclear localization of the transcription factor CrzA.

**Figure 6:**
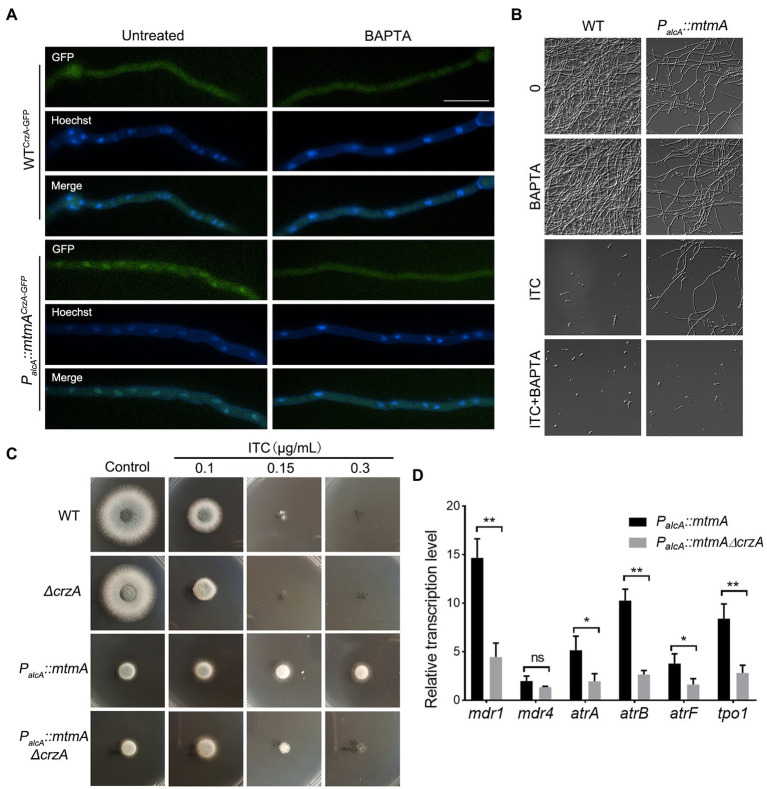
Repressed expression of MtmA causes persistent nuclear localization of CrzA, which contributes to drug resistance. **(A)** Fluorescence images of CrzA-GFP in mycelia under untreated or treated conditions with 30 μg/ml BAPTA for 30 min in the indicated strains. The merged images of GFP and Hoechst staining showed that CrzA-GFP was localized in the cytoplasm when treated with BAPTA. The scale bar represents 10 μm. **(B)** Germlings and hyphal growth phenotypes in the indicated strains under liquid MM treatment with BAPTA (30 μg/ml), ITC (0.5 μg/ml) or ITC combined with BAPTA at 37°C for 24 h. **(C)** Conidia of each indicated strain were inoculated on repression (YAG) medium containing serial concentrations of itraconazole (ITC) for 1.5 or 2 days at 37°C. **(D)** The transcript levels of the indicated genes in the indicated strains grown at 37°C for 24 h. Error bars indicate the mean ± SD of the results from three independent experiments. Statistical significance was determined by Student’s *t* test. *, *p* < 0.05; **, *p* < 0.01; ns, not significant.

To confirm whether the persistent nuclear localization of CrzA leads to multidrug resistance in the *mtmA*-repressed strain, we prevented the persistent nuclear localization of CrzA by adding the Ca^2+^ chelator 1,2-bis (2-aminophenoxy)-ethane -N,N,N′N′-tetraacetic acid (BAPTA). As shown in Figures 6A,B, the addition of BAPTA significantly decreased the cytoplasmic localization of CrzA-GFP in the *mtmA*-repressed strain. Accordingly, BAPTA also reduced the azole resistance of the *mtmA*-repressed strain under liquid culture conditions. To further verify whether the persistent nuclear localization of CrzA contributes to multidrug resistance in the *mtmA*-repressed strain, we constructed a deletion strain of CrzA in the background of the *P_alcA_::mtmA* strain. As shown in Figure 6C, deletion of CrzA significantly reduced the azole resistance of the *mtmA*-repressed strain, suggesting that repression of *mtmA* expression resulted in azole resistance dependent on the transcription factor CrzA. Moreover, RT–PCR results showed that the deletion of CrzA also significantly reduced the transcript levels of related drug pump genes in the *mtmA*-repressed strain (Figure 6D). In conclusion, our data suggest that the drug resistance involved in the *mtmA*-repressed strain is mainly related to the sustained nuclear localization of CrzA, which could be induced by elevated cytoplasmic Ca^2+^.

## Discussion

The resistance of fungal pathogens have steadily increased in recent years due to the long-term clinical and agricultural use of azoles, which poses a great challenge for the treatment of fungal diseases (Camps et al., 2012; Chowdhary et al., 2013; Verweij et al., 2020). In addition to resistance caused by mutational modifications of the azole target protein Erg11A in *A. fumigatus*, there is growing evidence from clinicians and researchers that complex and unknown resistance mechanisms exist (Fraczek et al., 2013; Hagiwara et al., 2018). In this study, we found that mitochondria-localized MtmA is involved in the resistance to multiple antifungal drugs.

As an organelle necessary for energy production in eukaryotic cells, mitochondria are involved in a series of metabolic processes, such as apoptosis, Ca^2+^ homeostasis, and lipid and other metabolite biosynthesis (Vakifahmetoglu-Norberg et al., 2017; Akbari et al., 2019; Rossi et al., 2019). Currently, much evidence suggests that the integrity of mitochondrial function plays an important role in susceptibility to azole antifungal drugs (Neubauer et al., 2015; Li et al., 2020; Zhou et al., 2022). Here, phenotypic analysis revealed that repression of MtmA expression resulted in significant resistance to the azole drug itraconazole (Figure 1). Our previous study found that the mitochondria-localized metal chaperone protein MtmA affects the mitochondrial membrane potential, which is critical for mitochondrial function, in addition to being important for the oxidative stress response and mycelial growth (Zhai et al., 2022). This suggests that the involvement of MtmA in itraconazole resistance may be caused by impaired mitochondrial function. Many studies have shown that the high expression of the azole target Erg11A and the content of its substrate ergosterol are important reasons for the development of azole resistance in fungal pathogens (Blatzer et al., 2011; Gsaller et al., 2016). Unexpectedly, Our data showed that repression expression of MtmA significantly reduced the expression level of Erg11A/B and ergosterol content (Figure 2), suggesting that the involvement of MtmA in azole resistance is not due to an altered drug target. According to a study in yeast, the deletion of Mtm1 reduced the transport of pyridoxal 5′-phosphate (PLP) for the function of 5-aminolevulinate synthase in the heme biosynthetic pathway, and therefore reduced heme biosynthesis (Whittaker et al., 2015). Thus, heme plays an important role for the function of Erg11. Therefore, this information suggests that reduced expression of MtmA in *A. fumigatus* affects expression of Erg11 probably through reducing heme synthesis.

Our RNA-seq data show that repressed expression of MtmA leads to the upregulation of a series of drug efflux pumps, particularly the drug efflux pump representative Mdr1 (Figure 3A). These data were further confirmed by RT–PCR and LacZ assays (Figures 3B–D). Consistent with this result, the HPLC assay showed that the repressed expression of MtmA significantly reduced the intracellular ITC content compared to the WT strains, implying that the involvement of MtmA in drug resistance is caused by reduced drug entrance. In fungi, stimulation by various external stresses activates the Ca^2+^ signaling pathway, leading to nuclear translocation of the transcription factor CrzA, which regulates downstream signaling pathways to alleviate cellular stress and promote cell survival (Juvvadi et al., 2014, 2017). Recent studies in *A. fumigatus* have shown that mitochondrial dysfunction can activate the Ca^2+^ signaling pathway and then upregulate a series of drug efflux pumps dependent on the transcription factor CrzA for their survival against antifungal drugs (Li et al., 2020). Interestingly, our RNA-seq and RT–PCR data showed that repressed expression of MtmA resulted in significant upregulation of the calcium transporter ATPase PmcC and that most of the upregulated drug efflux pump genes were consistent with the previously reported transcription factor CrzA-dependent drug efflux pump (Figure 3; Supplementary Figure S3), implying that MtmA may be involved in drug resistance through the calcium signaling pathway. Further studies revealed that CrzA was always localized in the nucleus regardless of calcium stimulation and repression of MtmA expression, suggesting that the abnormal calcium homeostasis resulting from repressed expression of MtmA may be related to the persistent nuclear localization of CrzA (Figure 5). Moreover, deletion of CrzA significantly reduced the transcript levels of azole resistance and the related drug efflux pumps involved in the repressed expression of MtmA. These data suggest that the CrzA-regulated calcium signaling pathway is involved in drug resistance due to the repressed expression of MtmA. However, repressed expression of MtmA is able to cause the reduced Erg11 protein expression, and then result in decreased ergosterol synthesis which is opposite with azole resistance phenotype, for which we speculate that was due to slowly growth rate accompanied with less requirement for ergosterol in the *mtmA*-repressed strain. Therefore, we conclude that the reduction in intracellular drug retention was the major contribution for resistance in the repressed expressed MtmA mutant.

Mitochondria are organelles necessary to provide energy for various biological processes and play an important role in buffering transient increases in cytoplasmic free calcium (Ganitkevich, 2003). MtmA may be an essential gene in *A. fumigatus*, and low expression leads to growth defects, a decreased metabolic rate and mitochondrial dysfunction (Zhai et al., 2022), resulting in abnormal activation of calcium signaling. In conclusion, we characterized a novel function of the metal chaperone protein MtmA involved in multiple antifungal drug resistance by affecting the CrzA-regulated calcium signaling pathway.

## Data availability statement

The datasets presented in this study can be found in online repositories. The names of the repository/repositories and accession number(s) can be found at: https://www.ncbi.nlm.nih.gov/, PRJNA891494.

## Author contributions

PZ: data curation, writing – original draft, visualization, and investigation. PZ, YM and WD: conceptualization and methodology. LL: writing – review and editing and supervision. All authors contributed to the article and approved the submitted version.

## Funding

This work was financially supported by the National Key R&D Program of China (2019YFA0904900) and the National Natural Science Foundation of China (NSFC) (grants 82172292 and 31861133014 to LL), and the Priority Academic Program Development (PAPD) of Jiangsu Higher Education Institutions.

## Conflict of interest

The authors declare that the research was conducted in the absence of any commercial or financial relationships that could be construed as a potential conflict of interest.

## Publisher’s note

All claims expressed in this article are solely those of the authors and do not necessarily represent those of their affiliated organizations, or those of the publisher, the editors and the reviewers. Any product that may be evaluated in this article, or claim that may be made by its manufacturer, is not guaranteed or endorsed by the publisher.

## Supplementary material

The Supplementary material for this article can be found online at: https://www.frontiersin.org/articles/10.3389/fmicb.2022.1062282/full#supplementary-material
