# Supplementary material for: The metal chaperone protein MtmA plays important roles in antifungal drug susceptibility in Aspergillus fumigatus
Source: Front Microbiol. 2022 Dec 2;13:1062282. doi: 10.3389/fmicb.2022.1062282 (PMC9755174; doi:10.3389/fmicb.2022.1062282)
Supplement: Supplementary file 2 [file Data_Sheet_1.doc]

Supplementary materials

*
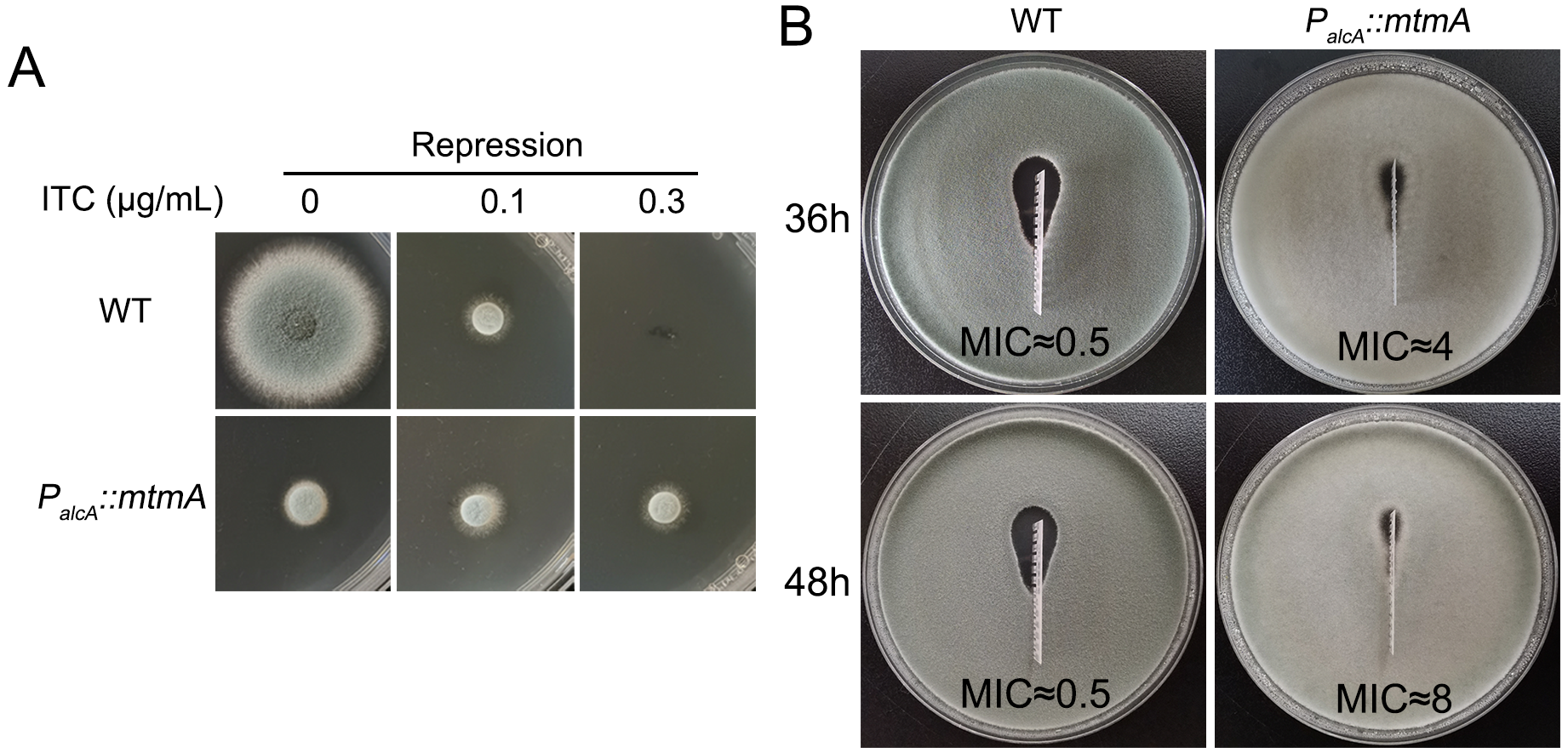
*

**Figure. S1** Repressed expression of MtmA causes itraconazole resistance. (A) Conidia of each indicated strain were inoculated on repression (YAG) medium containing serial concentrations of itraconazole (ITC) for 1.5 days at 37 °C. (B) The MIC test of the related strains was conducted using the commercialized E-test strip. Conidia (1 × 10^5^) of the WT or *P_alcA_:mtmA* were mixed in YAG, and then, the itraconazole test strip was placed on the plate and incubated at 37 °C for 36 h or 48 h.

*
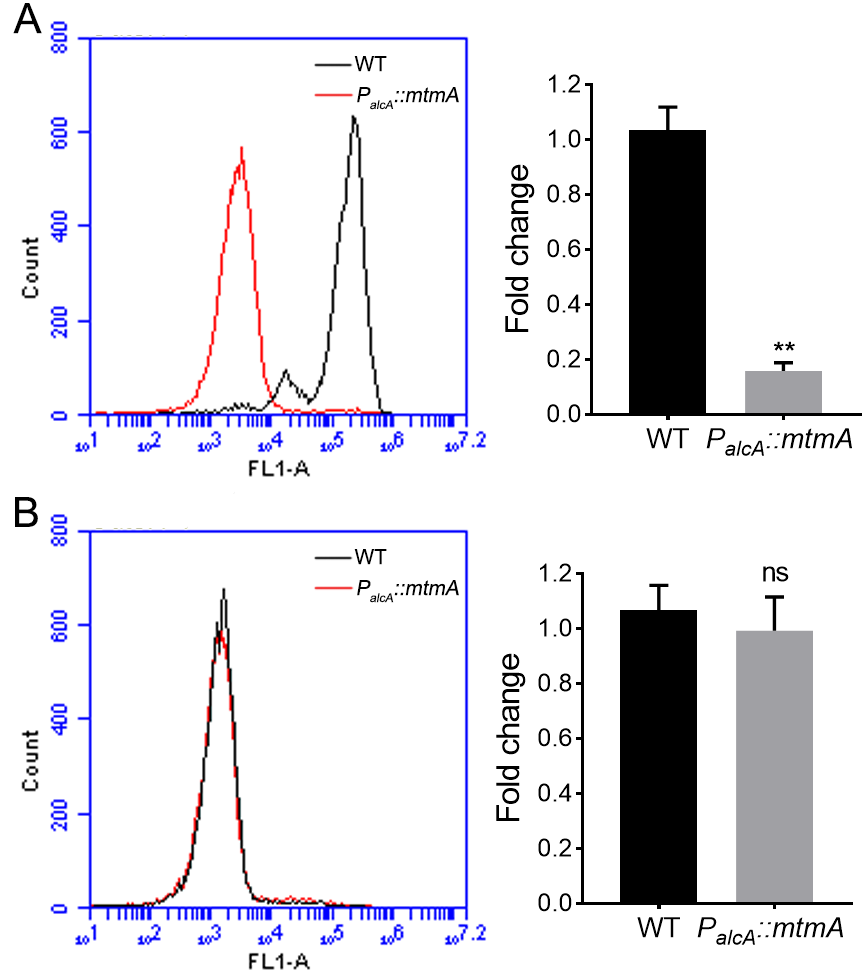
*

**Figure. S2** Flow cytometry analysis showing decreased R6G absorption in the *mtmA*-repressed strain. The uptake (A) and efflux (B) abilities of R6G were assessed in WT and *P_alcA_:mtmA* strain. FL1-A at the x axis represents the relative fluorescence intensity value. The fluorescence intensity was normalized to that of the parental strain under the same conditions. Error bars indicate the mean ± SD of the results from three independent experiments. **, *P* < 0.01.


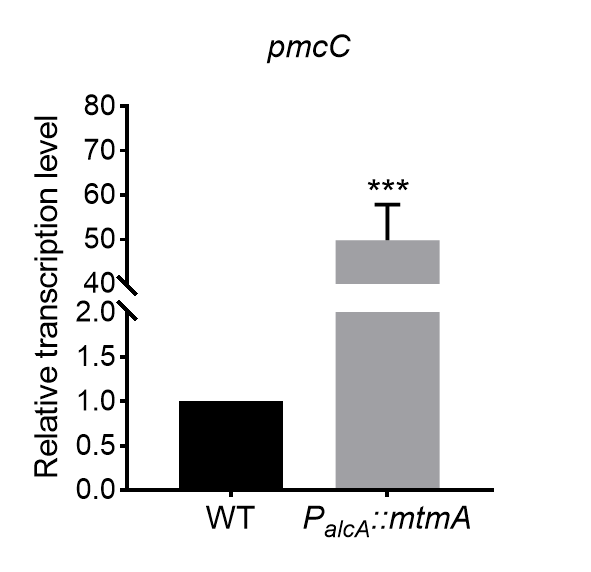


**Figure. S3** The relative expressions of the *pmcC* gene in the *P_alcA_::mtmA* strain was determined by real-time PCR. RT-qPCR analysis was performed after growth of cultures in liquid MM for 24h at 37℃. The *tubA* gene was used as an internal control. ***, *P* < 0.001, according to Student's *t* test.

**
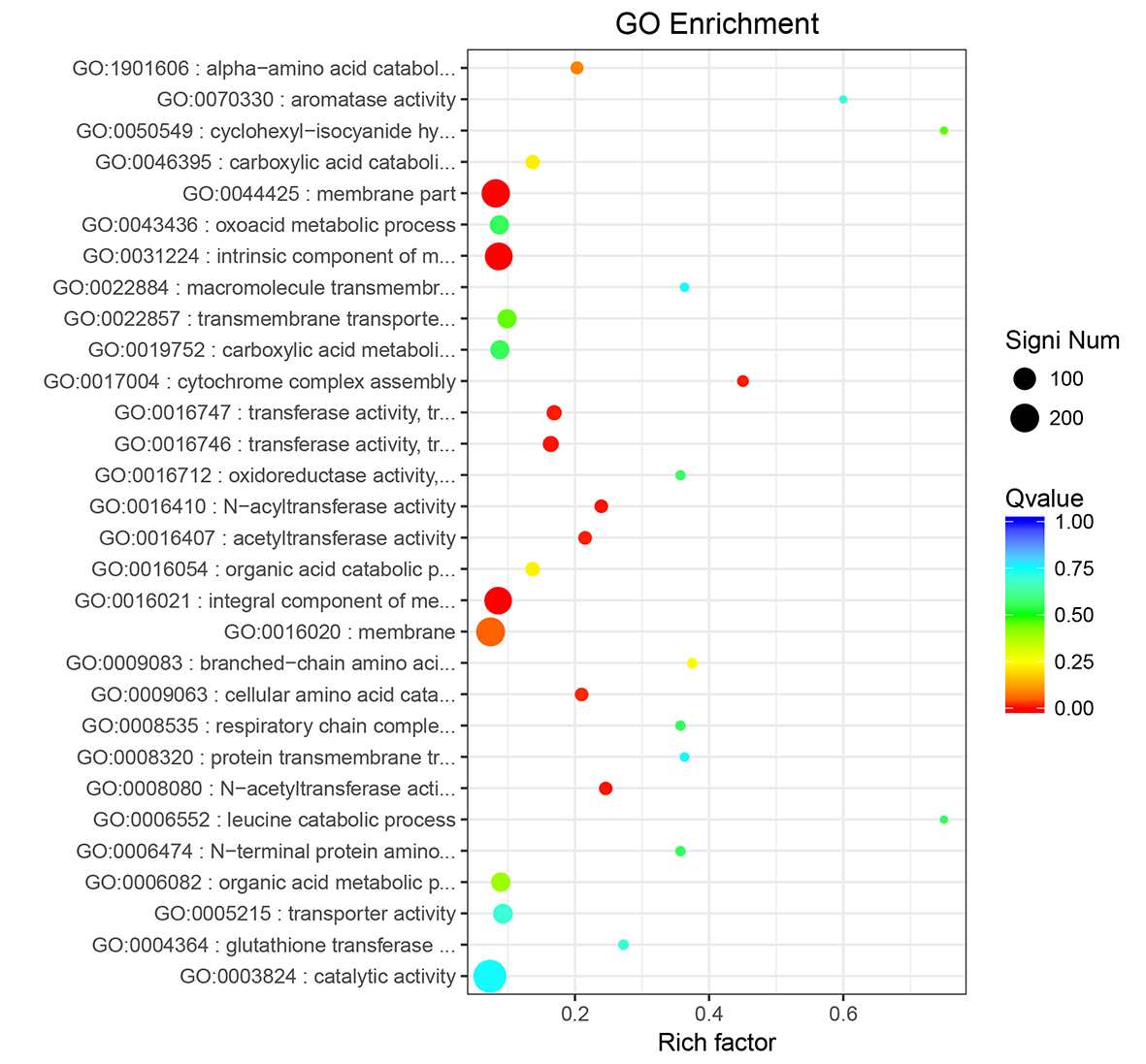
**

**Figure. S4** Functional category enrichment analyses were performed using GO in the *mtmA*-repressed strain versus WT strain.

**
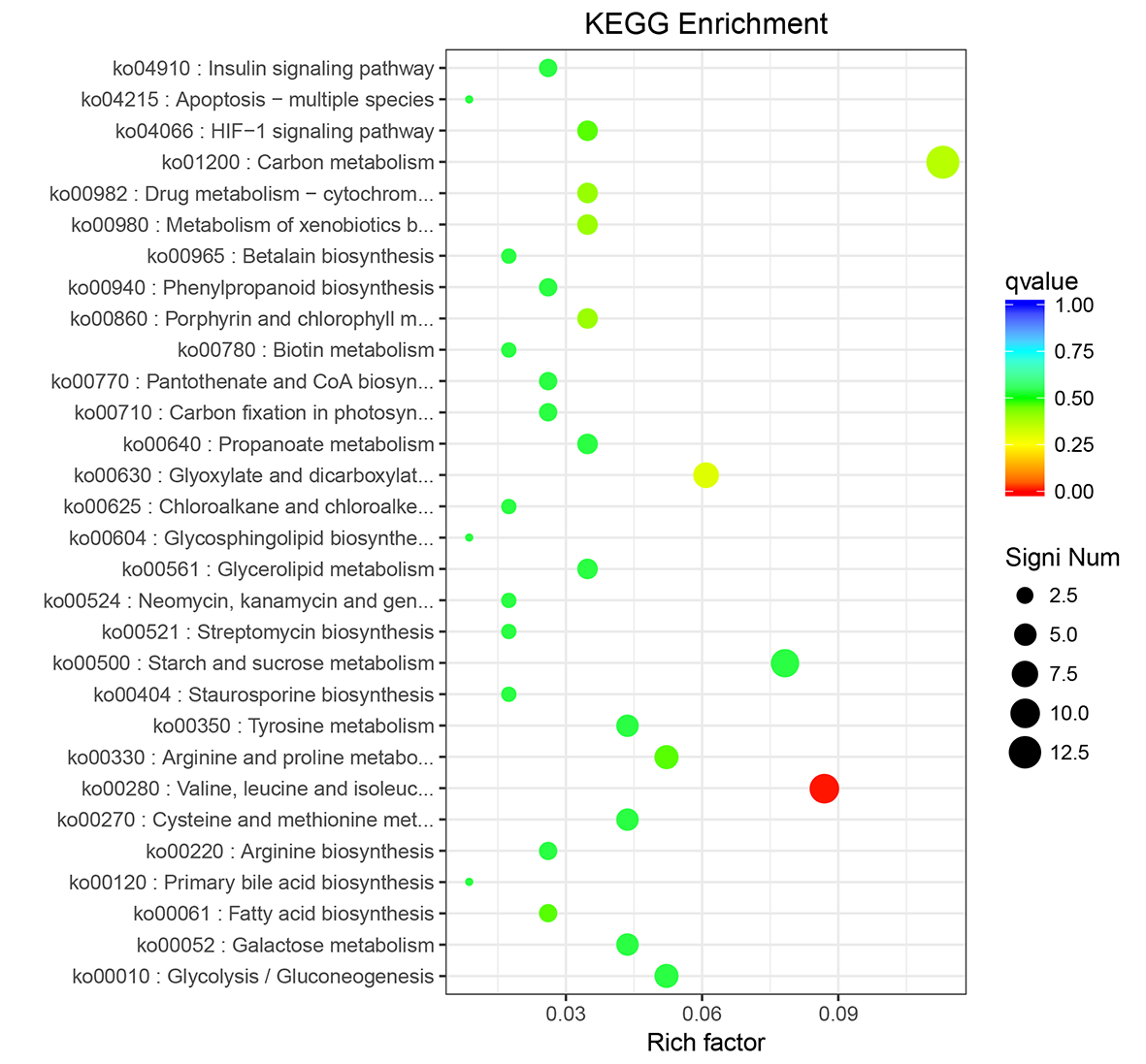
**

**Figure. S5** Signal pathway enrichment analyses were performed using KEGG databases in the *mtmA*-repressed strain versus WT strain.

**Table S1** ***Aspergillus fumigatus* strains used in this study**

| Strain | Genotype | Reference or source |
| --- | --- | --- |
| A1160 | *Δku80, pyrG* | FGSC |
| WT(A1161) | *Δku80,* *A1160:: pyrG* | (Jiang et al., 2014) |
| ZPF01 | *Δku80, pyrG, alc(p)-mtmA*::*pyr4* | (Zhai et al., 2022) |
| ZPF02 | *Δku80, pyrG, alc(p)-mtmA*::*pyr4, erg11A*::GFP::*hph* | This study |
| ZPF03 | *Δku80, pyrG, alc(p)-mtmA*::*pyr4, erg11B*::GFP::*hph* | This study |
| ZPF04 | *Δku80, pyrG, alc(p)-mtmA*::*pyr4, mdr1(p)::lacZ::hph* | This study |
| ZPF05 | *Δku80, A1160:: pyrG, mdr1(p)::lacZ::hph* | This study |
| ZPF06 | *Δku80, pyrG, alc(p)-mtmA*::*pyr4, AMA1::PgpdA::Aeq::hph* | This study |
| ZPF07 | *Δku80, A1160:: pyrG, AMA1::PgpdA::Aeq::hph* | This study |
| ZPF08 | *Δku80, pyrG, alc(p)-mtmA*::*pyr4, crzA*::GFP::*ptrA* | This study |
| ZPF09 | *Δku80, A1160:: pyrG,, crzA*::GFP::*ptrA* | This study |
| ZPF10 | *Δku80, pyrG, ΔcrzA*:: *pyr4* | This study |
| ZPF11 | *Δku80, pyrG, ΔcrzA*:: *pyr4, alc(p)-mtmA*::*hph* | This study |

**Table S2** **Primers used in this study**

| **Primer name** | **Sequence (5’ to 3’)** |
| --- | --- |
| RT-tub F | TTCCGTCCCGACAACTTCGT |
| RT-tub R | TCACAGCCTTCAGCCTCACG |
| RT-mdr1 F | CTGATCATACTCGTGAGCGC |
| RT-mdr1 R | CCAATACTTGACGTAGGCGA |
| RT-mdr4 F | GGTATCGTCTTCGGTGACCT |
| RT-mdr4 R | CACTCACCAAACACCAGCAA |
| RT-atrA F | GCATCCACGAGTCCAAGCGA |
| RT-atrA R | CCGCGCATATGCCAAGCATC |
| RT-atrB F | CTGGCCTCGACGGTCAATCC |
| RT-atrB R | TTGGCCAACAGCAACAGGGT |
| RT-atrF F | GCCGTCGTTCACCGTCATCT |
| RT-atrF R | AAGACCGGGTAGTCGCGGTA |
| RT-top1 F | ATGACTTTGGCTACCAGCCT |
| RT-top1 R | CATTCTGTGCCACTGCTACC |
| RT-pmcC F | TGCTGCGTCTGCTTCTCTCG |
| RT-pmcC R | CAGAGCGGCAAAGGTGTCCA |
| crzA-P1 | GGCAGAAACATTTCCTTGAT |
| crzA-P2 | GATAGTCAAACCCATGTCGA |
| crzA-P3 | ACACCCGCCAACACCCCACGTTGGTGAAACGTGATC |
| crzA-P4 | CGCCATTCGCCATTCATGAACCATCCGGTCAGATTC |
| crzA-P5 | CACGGACGAGCAGGACCTTC |
| crzA-P6 | CTCTTCCTGGACATCGGC |
| crzA-S | CCCAACAGGGACAGGAAA |
| crzA-A | GAGAAAGTGAGCGAGGAGTAG |

**References**

Jiang, H., et al., 2014. Deletion of the putative stretch-activated ion channel Mid1 is hypervirulent in Aspergillus fumigatus. Fungal Genet Biol. 62**,** 62-70.

Zhai, P. F., et al., 2022. Molecular Characterization and the Essential Biological Function of the Metal Chaperone Protein MtmA in Aspergillus fumigatus. Applied and Environmental Microbiology. 88.
